# Using Family Based Therapy (FBT) for older adolescents: strengths and difficulties

**DOI:** 10.1186/2050-2974-1-S1-O57

**Published:** 2013-11-14

**Authors:** Lisa McLeod, Kathryn Bell, Jennifer Lawrence

**Affiliations:** 1 Canberra Eating Disorders Program, ACT Health, Australia

## 

The Canberra Eating Disorders Program (EDP) is a specialised program which treats people with an eating disorder of any age. The EDP uses Family Based Therapy (FBT) to treat both younger and older adolescents with an eating disorder. FBT is the most effective treatment for adolescents with Anorexia Nervosa; however, the evidence supporting the use of FBT in the older adolescent and young adult group is mixed. There is also limited data around the experience of implementing FBT in treatment settings for the older adolescent age group. Anecdotal evidence suggests that treating older adolescents presents different challenges to treating younger adolescents and impacts clinical outcomes. Throughout treatment EDP has seen strengths using FBT in older adolescents including levels of engagement, recovery rates, and family involvement. However, there are also difficulties, such as acceptance of FBT as the treatment, and concerns around interrupting young adult development. Clinical outcomes at the EDP for the 16 to 19 year old age group suggests that FBT has a higher remission rate than individual therapy or day program, and leads to greater treatment adherence. However, in this older age group modification to the model is often required to improve treatment success.

This abstract was presented in the **Children and Youth Treatment and Service Development** stream of the 2013 ANZAED Conference.

